# The role of gut microbiota and plasma metabolites in ulcerative colitis: Insights from Mendelian randomization analysis

**DOI:** 10.1097/MD.0000000000041710

**Published:** 2025-02-28

**Authors:** XuWen Zheng, JinNuo Fan, JinNan Yin, Ying Chu

**Affiliations:** a Emergency Department, Wujin Hospital Affiliated with Jiangsu University and Wujin Clinical College of Xuzhou Medical University, Changzhou, Jiangsu, China; b Wujin Institute of Molecular Diagnostics and Precision Cancer Medicine of Jiangsu University, Wujin Hospital Affiliated with Jiangsu University, Changzhou, Jiangsu, China.

**Keywords:** gut microbiota, Mendelian randomization, meta-analysis, plasma metabolites, ulcerative colitis

## Abstract

Emerging research suggests that alterations in gut microbiota composition may play a significant role in the pathogenesis of ulcerative colitis (UC). Plasma metabolites, which are influenced by gut microbiota, have also been implicated, but their role in UC remains unclear. This study aims to determine whether specific plasma metabolites mediate the causal relationship between gut microbiota and UC using Mendelian randomization (MR) analysis. This study employed publicly available summary-level data from genome-wide association studies and metagenomic datasets. Gut microbiota data were derived from the FINRISK cohort (5959 participants), plasma metabolite data from the Canadian Longitudinal Study on Aging (8299 individuals), and UC data from multiple consortia (17,030 cases and 883,787 controls). Forward and reverse MR analyses, supplemented by linkage disequilibrium score regression (LDSC), were conducted to assess causal relationships. Mediation effects of plasma metabolites between gut microbiota and UC were analyzed using the product of coefficients method. Various sensitivity analyses, including MR-Egger and MR-PRESSO, were applied to detect pleiotropy and ensure robust results. The study identified 20 bacterial taxa and 93 plasma metabolites linked to UC. Forward MR analysis showed that *Clostridium S felsineum* increased UC risk via reduced carnitine levels, with a mediation proportion of 39.77%. *Eubacterium callanderi* was associated with decreased UC risk through the tryptophan to pyruvate ratio (16.02% mediation). Additionally, species *CAG-590 sp000431135* increased UC risk through elevated mannitol/sorbitol levels, mediating 28.38% of the effect. Sensitivity analyses confirmed the robustness of these findings, with minimal heterogeneity and pleiotropy detected. This study highlights the significant role of gut microbiota and their associated plasma metabolites in the pathogenesis of UC. Specific microbial species influence UC through metabolites, suggesting potential therapeutic targets. Modulating carnitine, tryptophan metabolism, or sugar alcohols could offer promising avenues for UC management.

## 1. Introduction

Ulcerative colitis (UC) is a chronic inflammatory bowel disease (IBD) primarily affecting the colon and rectum. Epidemiologically, UC has been on the rise globally, with higher prevalence noted in developed countries, although recent trends show increasing incidence in newly industrialized nations as well.^[1]^ Clinically, UC is characterized by symptoms such as bloody diarrhea, abdominal pain, urgency, and rectal bleeding, often with periods of exacerbation and remission.^[2]^ In severe cases, extraintestinal manifestations, such as sclerosing cholangitis, may also occur.^[3]^ The societal and human toll of UC is profound. The chronic nature of UC, characterized by lifelong symptoms and frequent hospitalizations, significantly reduces patients’ quality of life and ability to work, resulting in high healthcare costs and emotional burdens.^[4]^

The pathogenesis of UC remains multifactorial, involving a complex interplay of genetic predisposition, immune system dysregulation, environmental factors, and gut microbiota imbalance.^[5]^ Recent advances in microbiome research have highlighted a crucial role of gut microbiota dysbiosis in IBD, including UC. Observational studies using 16S rRNA sequencing and metagenomics have consistently reported altered microbial diversity in UC patients, characterized by a decrease in beneficial commensals (e.g., *Firmicutes*) and an increase in pro-inflammatory bacteria such as *Proteobacteria*.^[6]^ Mendelian randomization (MR) studies provide strong evidence for a causal relationship between certain gut microbial genera and UC. For instance, MR analyses have identified a protective role for *Eubacterium ventriosum* in UC and a positive association between *Coprococcus 2* and increased UC risk.^[7]^ Despite these insights, the precise mechanistic pathways by which gut microbiota influence UC remain poorly understood, especially regarding how microbial activity affects host metabolic processes.

Gut microbiota exert significant effects on the host through the production and modulation of plasma metabolites, which serve as key mediators in host-microbiome interactions.^[8]^ Metabolites such as short-chain fatty acids (SCFAs), bile acids, amino acids, and sugar alcohols are known to regulate immune responses, epithelial integrity, and intestinal inflammation. However, it remains unclear which specific plasma metabolites mediate the causal relationship between gut microbiota and UC. Understanding these intermediary pathways is crucial, as metabolites represent potential biomarkers for UC risk stratification and therapeutic targets for microbiota-based interventions.

To address this gap, we employ a mediation MR analysis to investigate whether plasma metabolites mediate the causal relationship between gut microbiota and UC. MR is a genetic instrumental variable approach that helps establish causal relationships while minimizing confounding and reverse causation.^[9]^ By leveraging genome-wide association study (GWAS) data from large cohorts, we aim to: identify gut microbiota taxa causally associated with UC, determine plasma metabolites linked to UC, and evaluate the mediation effects (ME) of these metabolites in the gut microbiota–UC axis. Our findings will provide insights into how gut microbiota contribute to UC pathogenesis through metabolic intermediates, potentially paving the way for metabolite-targeted interventions.

## 2. Materials and methods

### 2.1. Study design

**Figure 1.**
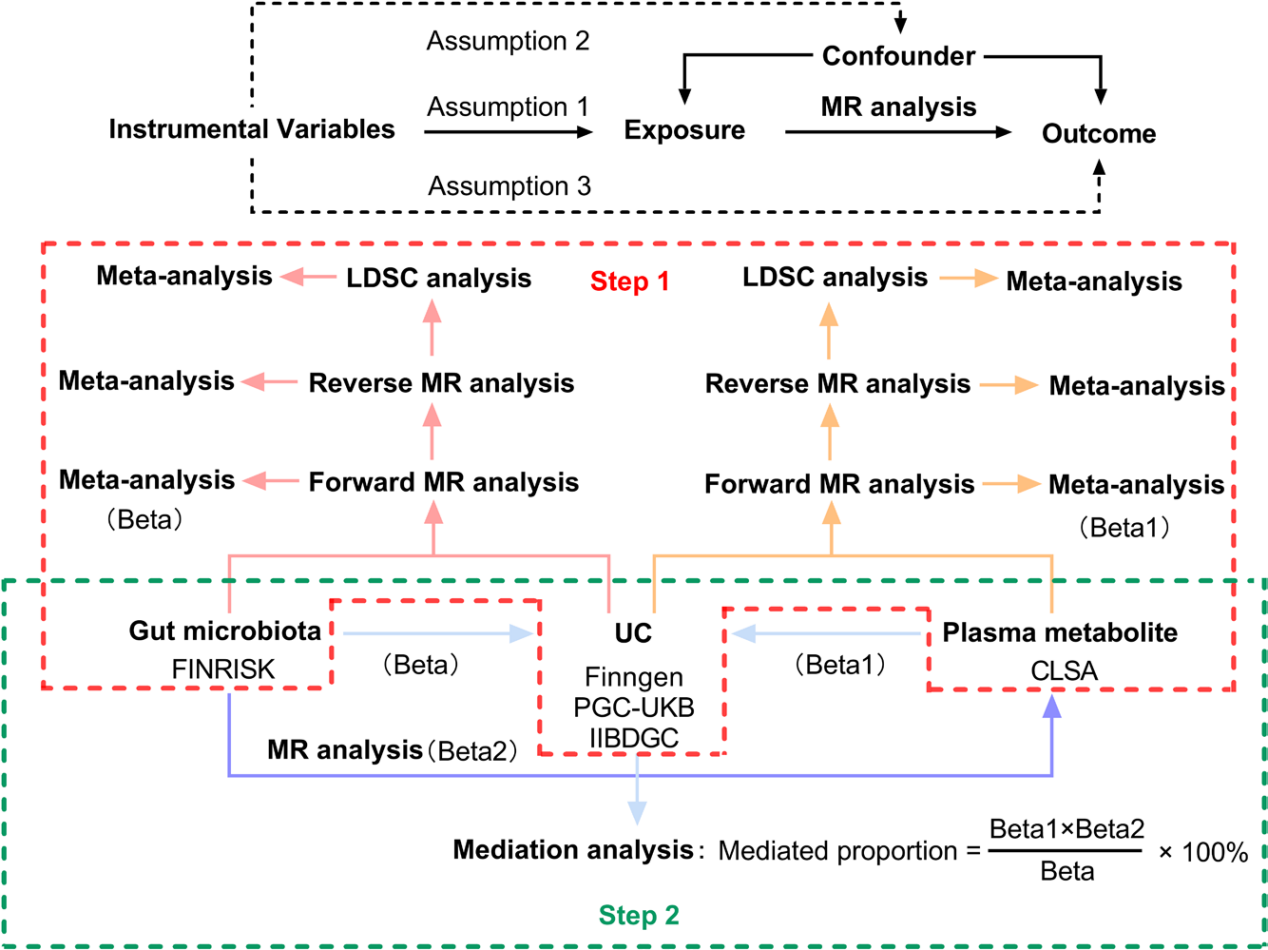
Three assumptions of MR analysis and overview of the study design. LDSC = linkage disequilibrium score regression, MR = Mendelian randomization, UC = ulcerative colitis.

All summary-level data used in our analysis are publicly accessible. The design of this study is illustrated in Figure 1. Initially, we explored the relationship between gastrointestinal microbiomes, plasma metabolites, and UC using forward and reverse MR, and linkage disequilibrium (LD) score regression (LDSC) analyses. We integrated the results from these MR and LDSC analyses in a meta-analysis to comprehensively evaluate UC using diverse data sources. In the second phase, we compared the gastrointestinal microbiomes identified previously with the associated plasma metabolites. We applied the product of coefficients method to assess the ME of these microbiomes on UC mediated by plasma metabolites.^[10]^ Our analytical procedures adhered to the STROBE-MR guidelines.^[11]^ Ethical approval was secured for all included studies, and consent was obtained from all participants, in compliance with the guidelines of their institutional review boards or ethical committees respectively.

Summary-level data on gut microbiota were obtained from the FINRISK study, which involved 5959 participants who had been genotyped and had their gut microbial metagenomes analyzed, resulting in the identification of 473 unique taxa.^[12]^ Additionally, GWAS encompassing 1091 metabolites and 309 metabolite ratios were derived from the Canadian longitudinal study on aging (CLSA), which involved 8299 individuals of European descent.^[13]^ Information related to UC was compiled from several key sources, including the Pan-UKB GWAS Version 0.4, released March 16, 2023;^[14]^ the FinnGen GWAS Release 10, released December 18, 2023;^[15]^ and the International IBD Genetics Consortium (IIBDGC).^[16]^ These sources contributed to a combined sample of 17,030 cases and 883,787 controls, all of European descent. Detailed diagnostic criteria, adjustments, and sample sizes are presented in Table 1. An online tool was used to estimate the power of the MR analysis.^[17]^ Given that our sample data were sourced from 5 distinct databases, we effectively mitigated the risk of sample overlap.

**Table 1:** Detailed information on used summary-level data.

Exposure or outcome	Unit	Consortium	Participants included in analysis	Age (yr)	Male (%)	Adjustments	ICD	PMID	Web source
Gut microbiota	SD	FINRISK	5959 European individuals	45.7 ± 11.5	50	Age, sex, genotyping batch and top ten genetic principal components		35,115,689	https://www.ebi.ac.uk/gwas/
Plasma metabolite	SD	CLSA	8299 European individuals	63 (45–85)	52	Age, sex, hour since last meal or drink, genotyping batch and the first ten genetic principal components		36,635,386	https://www.ebi.ac.uk/gwas/
Ulcerative colitis	One-unit in log-transformed odds ratio of ulcerative colitis	FinnGen	6435 cases and 446,419 controls of European ancestry	53 ± 18	44	Sex, age, genotyping batch and ten principal components	ICD-10: K51	36,653,562	https://r10.finngen.fi/
		Pan-UKB	3627 cases and 416,904 controls of European ancestry	55.1 ± 7.6	48	Sex, age, genotyping array, and the first 8 principal components	ICD-10: K51		https://pan.ukbb.broadinstitute.org/downloads/
		IIBDGC	6968 cases and 20,464 controls of European ancestry	34.1 ± 15.8	52	7 principal components		26,192,919	https://www.ibdgenetics.org/

Abbreviations: CLSA = Canadian longitudinal study on aging, IIBDGC = International Inflammatory Bowel Disease Genetics Consortium.

To conduct MR analysis, IVs must meet 3 essential criteria: the selected genetic variants should be robustly linked to the exposure being studied; these variants must be independent of other factors that could influence the outcome to avoid confounding; and the relationship between the genetic variants and the outcome should occur exclusively through the exposure in question.^[9]^ When using a threshold of *P* < 5 × 10^−8^ and applying a stringent LD clumping setting with a 10,000 kb distance and r^2^ < 0.001 between IVs, we found that only 284 bacterial taxa and 839 plasma metabolites had total of 324 and 2230 single nucleotide polymorphisms (SNPs) meeting these criteria respectively. To satisfy the first MR analysis assumption, the exposure identified by GWAS was subjected to a threshold of *P* < 5 × 10^−6^.^[18]^ For each SNP, the *F*-statistic was calculated using the formula *F* = beta²/se², ensuring that weak IVs had a reduced influence. SNPs with an *F*-statistic lower than 10 were excluded from the analysis.^[19,20]^ To ensure consistency, the effect alleles were aligned between the exposure and outcome datasets, excluding any mismatched alleles. Furthermore, ambiguous palindromic SNPs with a minor allele frequency (MAF) close to 0.5 were also discarded to avoid errors. Proxy SNPs were not employed to substitute for missing IVs, as a small percentage of instruments were absent and had minimal impact on the results. In order to detect pleiotropy, we implemented MR pleiotropy residual sum and outlier (MR-PRESSO) test and MR-Egger intercept test. We excluded MR estimates that exhibited significant horizontal pleiotropy from the meta-analysis. Lastly, in order to maintain the third MR postulate, SNPs that were significantly linked to outcome (*P* < 5 × 10^−6^) were excluded. After the initial IV selection, the forward MR analyses included 1178 plasma metabolites and 416 bacterial taxa. Table S1 and S2, Supplemental Digital Content, http://links.lww.com/MD/O457 contains a comprehensive inventory of IVs that are associated with all bacterial taxa and plasma metabolites.

### 2.2. Statistical analysis

Under the random-effects framework, the main MR estimates were calculated using the inverse-variance weighted (IVW) method. When all genetic variants meet the fundamental MR assumptions, the most precise estimates can be obtained because the IVW method is most efficient in the absence of horizontal pleiotropy.^[9]^ Additionally, 3 sensitivity analyses were conducted: the weighted median, MR-Egger, and MR-PRESSO. When directional pleiotropy is suspected, MR-Egger is highly valuable, as it can test for pleiotropy through the intercept term and provide more conservative estimates. A significant intercept value indicates the presence of directional pleiotropy.^[21]^ The weighted median method is less susceptible to invalidity than the IVW method and provides more reliable estimates, especially suitable when minor pleiotropy exists.^[22]^ If pleiotropy is present or suspected, MR-PRESSO enhances the accuracy of causal estimations by excluding the impact of outlier variations that violate the assumption of exclusion limitation.^[23]^ Cochran *Q* test was applied to assess heterogeneity among the SNPs. To further investigate horizontal pleiotropy, the MR-Egger intercept test was performed. SNPs with a *P*-value of <.05 in either the intercept or global test, indicating significant pleiotropy, as well as cases involving fewer than 4 SNPs, were omitted from the meta-analysis because MR-PRESSO requires a minimum of 4 SNPs. Finally, the estimates from the IVW method and sensitivity analyses were synthesized using a fixed-effects meta-analysis. However, IVW results showing significant heterogeneity or inconsistencies with sensitivity analyses were excluded. We used LDSC to study the genetic correlation between UC and previously identified gut microbiota. Using HapMap3 as reference, non-SNP and SNPs with a MAF below 0.01, duplications, and SNPs with ambiguous strand orientation were excluded from the GWAS data. LDSC effectively identifies genetic correlations by analyzing the links between LD and test statistics.^[24]^ Genetic correlation is determined by multiplying the *z*-values of the variants for 1 trait with the z-values for another trait and then regressing these products on LD scores.^[25]^

A 2-step MR was conducted to perform mediation analysis.^[26]^ To streamline the process, we firstly examined the relationship between bacteria, metabolites and UC, followed by investigating the association between identified gastrointestinal microbiota and plasma metabolites. The product of coefficients method was applied to estimate the effect of microbiota on UC mediated by metabolites.^[10]^ ME was divided by the total effect to determine the mediation proportion.^[27]^

To control the false discovery rate, Bonferroni correction was applied in the meta-analyses of MR studies.^[28]^ For gut microbiota and plasma metabolites respectively, causal associations were considered significant when IVW *P*-values were <1.20 × 10^−4^ (.05/416) and 4.24 × 10^−5^ (.05/1178), and were considered suggestive if IVW *P*-values were between the abovementioned thresholds and .05. The TwoSampleMR and meta packages were employed using R software (version 4.3.1) for all the analyses.

## 3. Results

### 3.1. Gut microbiota and UC

**Figure 2.**
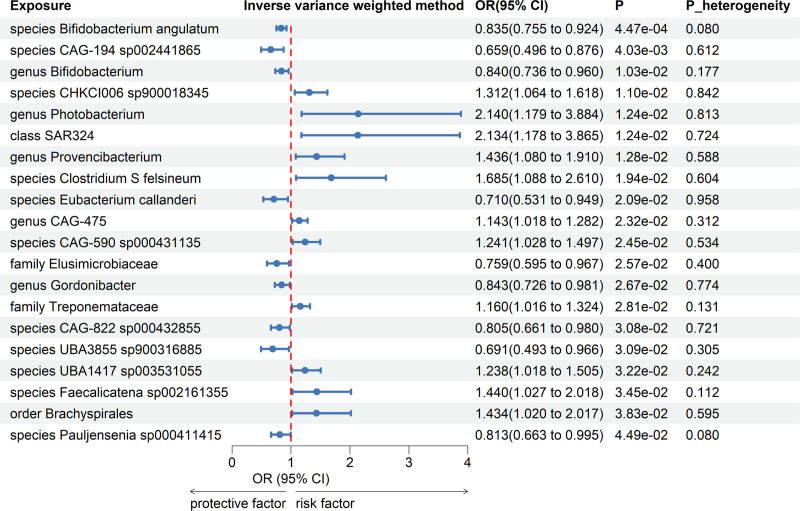
Forest plot of forward MR analysis between gut microbiota and ulcerative colitis. CI, confidence interval, MR = Mendelian randomization, *P*_heterogeneity = *P*-value of heterogeneity for meta-analysis, OR = odds ratio.

**Figure 3.**
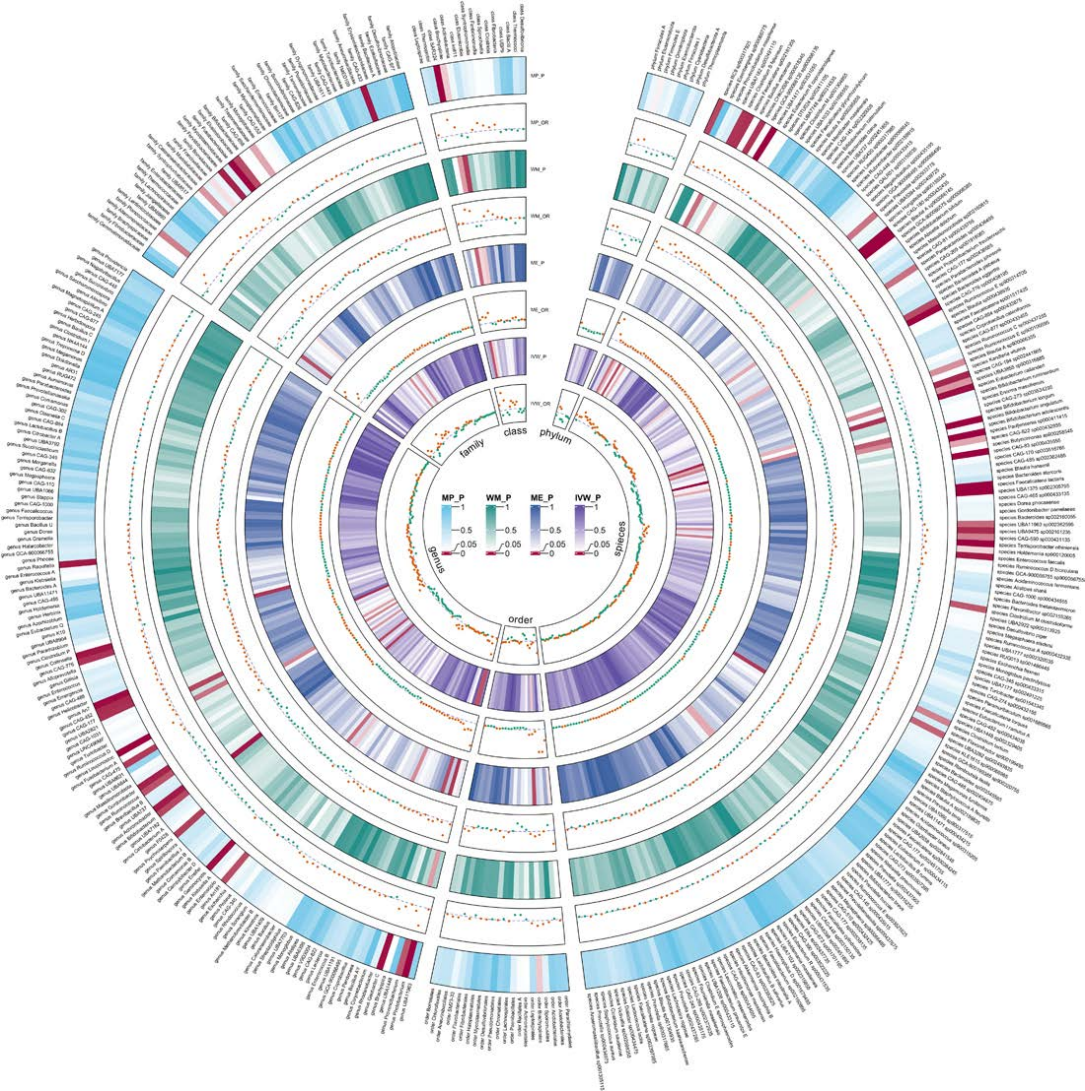
Circular heat map of meta-analysis of genetic correlation between gut microbiota and ulcerative colitis. The color variations represented the size of the *P*-value. The scatter plots reflect OR, with OR > 1 labeled red and OR < 1 labeled green. IVW = inverse-variance weighted, ME = MR-Egger, MP = MR-PRESSO, WM = weighted median.

Meta-analyses of 416 bacterial taxa were conducted (Table S3, Supplemental Digital Content, http://links.lww.com/MD/O457). Finally, we identified 20 gut microbiomes suggestively linked to UC. The combined IVW estimates showed that genetically predicted species *Clostridium S felsineum* (OR = 1.685, 95% CI = 1.088–2.610; *P* = .02), species *CAG-590 sp000431135* (OR = 1.241, 95% CI = 1.028–1.497; *P* = .02), and 9 other gut microbiomes were potentially linked to an increased risk of UC. Furthermore, genetically predicted species *Eubacterium callanderi* (OR = 0.710, 95% CI = 0.531–0.949; *P* = .02) and 8 other gut microbiomes were potentially linked to a decreased risk of UC (Fig. 2). All main results and sensitivity analyses were depicted in Figure 3.

**Figure 4.**
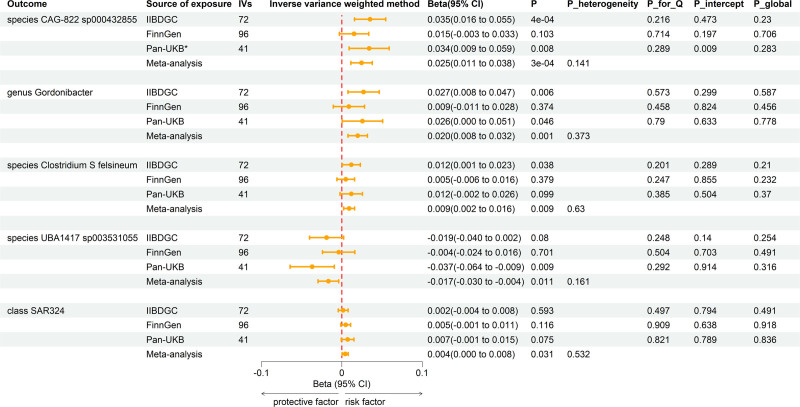
Forest plot of reverse MR analysis between identified gut microbiota and ulcerative colitis. *, excluded from the meta-analysis due to SNPs <4 or significant pleiotropy. CI = confidence interval; IVs = instrumental variables, MR = Mendelian randomization, *P*_heterogeneity = *P*-value of heterogeneity for meta-analysis, *P*_Q = *P*-value for Cochran *Q* test, *P*_intercept = *P*-value for MR-Egger intercept test, *P*_global = *P*-value for global test.

Reverse MR analyses were conducted between UC and previously identified 20 gut microbiomes (Table S4, Supplemental Digital Content, http://links.lww.com/MD/O457). The combined IVW estimates revealed that genetically predicted UC was potentially linked to the abundance of species *Clostridium S felsineum* (Beta = 0.009, 95% CI = 0.002–0.016; *P* = .01) and 4 other gut microbiomes (Fig. 4).

Due to low heritability and small sample sizes, only 16 previously identified gut microorganisms were suitable for LDSC analysis. The combined LDSC estimates showed a potential positive correlation between genetically predicted species *Clostridium S felsineum* (Rg = 0.580, Rg_Se = 0.169, Rg_*P* = .001), species *Eubacterium callanderi* (Rg = 1.071, Rg_Se = 0.517, Rg_*P* = .038), species *CAG-590 sp000431135* (Rg = 0.728, Rg_Se = 0.369, Rg_*P* = .048), and UC (Table 2). Detailed results of all genetic associations are provided in Table S5, Supplemental Digital Content, http://links.lww.com/MD/O457.

**Table 2:** Meta-analysis of genetic correlation between identified gut microbiota and ulcerative colitis from 3 large databases.

Exposure	Rg	Rg_Se	Rg_*P*	*P*_heterogeneity
species *Clostridium S felsineum*	0.580	0.169	.001	.492
species *CHKCI006 sp900018345*	0.203	0.092	.028	.621
species *Eubacterium callanderi*	1.071	0.517	.038	.411
family *Elusimicrobiaceae*	0.446	0.217	.040	.240
species *CAG-590 sp000431135*	0.728	0.369	.048	.396

### 3.2. Plasma metabolites and UC

Table S6, Supplemental Digital Content, http://links.lww.com/MD/O457 displays the results of the forward MR study, identifying 93 plasma metabolites genetically predicted to have a potential causal link to UC. Of these, genetically predicted carnitine levels (OR = 0.784, 95% CI = 0.685–0.898; *P* = 4.36 × 10^−4^) was causally linked with a decreased risk of UC, and tryptophan to pyruvate ratio (OR = 1.120, 95% CI = 1.036–1.211; *P* = .005) and mannitol/sorbitol levels (OR = 1.115, 95% CI = 1.016–1.223; *P* = .022) were causally linked with an increased risk of UC.

Reverse MR analyses were conducted between UC and previously identified 93 plasma metabolites (Table S7, Supplemental Digital Content, http://links.lww.com/MD/O457). The combined IVW estimates revealed that genetically predicted UC was potentially linked to the arachidonate to linoleate ratio (Beta = −0.023, 95% CI =  − 0.043 to −0.004; *P* = .020) and 5 other plasma metabolites.

Due to low heritability and small sample sizes, only 80 previously identified plasma metabolites were suitable for LDSC analysis (Table S8, Supplemental Digital Content, http://links.lww.com/MD/O457). The combined LDSC estimates showed a potential correlation between genetically predicted UC and the glucose to mannose ratio (Rg = 0.278, Rg_Se = 0.093, Rg_*P* = .003), and 5 other plasma metabolites (Table 3).

**Table 3:** Meta-analysis of genetic correlation between plasma metabolites and ulcerative colitis from 3 large databases.

Exposure	Rg	Rg_Se	Rg_*P*	*P*_heterogeneity
Glucose to mannose ratio	0.278	0.093	.003	.417
Phosphate to mannose ratio	0.212	0.077	.006	.868
Mannose levels	−0.175	0.069	.011	.443
Adenosine 5’-monophosphate to glycine ratio	−0.217	0.095	.022	.204
Behenoyl sphingomyelin levels	−0.217	0.103	.035	.875
3-aminoisobutyrate levels	0.222	0.112	.047	.912

### 3.3. Mediation analysis

One thousand eight hundred sixty MR analyses (20 × 93) were conducted to examine the relationships between the identified bacterial taxa and plasma metabolites (Table S9, Supplemental Digital Content, http://links.lww.com/MD/O457). A total of 34 pairs of gut microbiomes and plasma metabolites were included in mediation analyses (Table S10, Supplemental Digital Content, http://links.lww.com/MD/O457). Specifically, the species *Clostridium S felsineum* indirectly influenced UC through carnitine levels, with an ME of 0.208 (95% CI = 0.044–0.371, *P* = .013), the species *Eubacterium callanderi* indirectly influenced UC through tryptophan to pyruvate ratio, with an ME of − 0.055 (95% CI =  − 0.108 to −0.002, *P* = .042), and the species *CAG-590 sp000431135* indirectly influenced UC through mannitol/sorbitol levels, with an ME of 0.061 (95% CI = 0.001–0.122, *P* = .047). The mediated proportions were 39.77%, 16.02%, and 28.38%, respectively (Table 4).

**Table 4:** Mediation analysis between gut microbiota, plasma metabolites, and ulcerative colitis.

Gut microbiota	Metabolite	Total effect EO (95% CI)	Effect EM (95% CI)	Effect MO (95% CI)	Mediation effect (95% CI)	Mediated proportion
species *Clostridium S felsineum*	Carnitine C14 levels	0.5218 (0.0844–0.9592)	−0.8534 (−1.3311, −0.3757)	−0.2432 (−0.3787 to −0.1077)	0.2075 (0.0436 to 0.3714) *P* = .0131	39.77%
species *Eubacterium callanderi*	Tryptophan to pyruvate ratio	−0.342 (−0.6321 to −0.0518)	−0.483 (−0.8058 to −0.1601)	0.1134 (0.035 to 0.1918)	−0.0548 (−0.1075 to −0.0021) *P* = .0416	16.02%
species *CAG-590 sp000431135*	Mannitol/sorbitol levels	0.2156 (0.0278 to 0.4034)	0.5635 (0.2853 to 0.8417)	0.1087 (0.0159 to 0.2015)	0.0612 (0.0008 to 0.1216) *P* = .0469	28.38%

Abbreviations: EM = exposure to mediator, EO = exposure to outcome, MO = mediator to outcome.

### 3.4. Sensitivity analysis, pleiotropy, and heterogeneity

During data processing, we excluded IVW estimates that were inconsistent with the sensitivity analysis (weighted median, MR-Egger, and MR-PRESSO) or exhibited significant pleiotropy (*P* for intercept < .05 or *P* for global test < .05) to maintain the stability and validity of our findings. Most SNPs (Cochran *Q* test) and meta-analysis results showed no or mild heterogeneity, demonstrating the robustness of our analyses.

## 4. Discussion

The study employed MR to investigate the causal relationships between gut microbiota, plasma metabolites, and UC. Forward MR analyses identified 20 bacterial taxa and 93 plasma metabolites associated with UC. The mediation analysis identified several significant indirect effects of gut microbiota on UC through plasma metabolites. Specifically, *Clostridium S felsineum* influenced UC indirectly via carnitine levels, with a mediation proportion of 39.77%. Additionally, *Eubacterium callanderi* affected UC through the tryptophan to pyruvate ratio with a mediation proportion of 16.02%. Furthermore, *CAG-590 sp000431135* influenced UC through mannitol/sorbitol levels with a mediation proportion of 28.38%. These findings highlight key microbial species and metabolites involved in the pathogenesis of UC.

More than 90% of the makeup of the gut microbiota community is accounted for by the phyla *Firmicutes*, *Bacteroidetes*, *Proteobacteria*, and *Actinobacteria*.^[29]^ The multifaceted effects of gut microbiota have indeed shown compelling evidence linking its dysregulation, known as dysbiosis, to a spectrum of pathologies, including dyslipidemia, insulin resistance, systemic inflammation, and IBD.^[30–33]^ Research conducted in Polish UC patients confirmed that dysbiosis results in a significant decrease in microbial diversity, with increased levels of *Proteobacteria* and *Actinobacteria*, both associated with inflammation, and a reduction of anti-inflammatory bacteria like *Bacteroidetes*.^[34]^ Emerging therapeutic approaches target gut dysbiosis, including fecal microbiota transplantation and probiotic treatments. These aim to restore a balanced microbiota, with mixed success in UC patients. More standardized treatments are needed to establish efficacy.^[35]^ Other studies have shown that restoring the gut microbiota through probiotics, such as *Lactobacillus rhamnosus* and *Bifidobacterium bifidum*, can modulate inflammation and improve UC symptoms, by increasing SCFAs and promoting immune regulation.^[36,37]^ In conclusion, dysbiosis plays a crucial role in the pathogenesis of UC, and therapeutic strategies targeting the gut microbiota hold potential in alleviating the disease.

Species *Clostridium S felsineum*, including members from the *Clostridiales* order, have been shown to influence various metabolic pathways, including the breakdown and utilization of carnitine. Carnitine is metabolized by gut bacteria into metabolites such as trimethylamine, which is then further processed in the liver to form trimethylamine-N-oxide, a compound linked to cardiovascular disease. However, in certain bacterial populations, such as those involving *Clostridium* species, carnitine can also be processed in a way that reduces its availability, impacting circulating carnitine levels and fatty acid transport within the body.^[38]^ The decrease in carnitine levels, a specific long-chain acylcarnitine, can be attributed to how certain gut bacteria like *Clostridium S felsineum* participate in the retroconversion of carnitine-related compounds, reducing their availability in the bloodstream. This aligns with the microbial pathways identified for *Clostridium* species that are involved in modifying host carnitine metabolism, resulting in altered levels of carnitine-related compounds like C14-carnitine.^[39]^ Our study revealed that the species *Clostridium S felsineum* could increase UC risk through decreasing carnitine levels. Decreased expression of carnitine transporters like organic cation/carnitine transporter 2 led to reduced carnitine uptake. This deficiency impaired the β-oxidation of butyrate, a key energy source for colonocytes. Carnitine supplementation helped restore normal butyrate metabolism and reduced colonic inflammation, suggesting that carnitine depletion plays a key role in UC pathogenesis.^[40]^ Oxidative stress is also a major contributor to tissue damage in UC. L-carnitine supplementation has shown promise in reducing oxidative stress markers, such as malondialdehyde and myeloperoxidase, in colitis models, which could mitigate tissue damage and inflammation in the colon.^[41]^ In summary, the interaction between *Clostridium S. felsineum* and carnitine metabolism may contribute to UC by disrupting essential metabolic processes and exacerbating inflammation.

In *Eubacterium callanderi*, the production of volatile fatty acids and its ability to demethoxylate aromatic compounds suggests its involvement in similar metabolic pathways where tryptophan could be used as a precursor for pyruvate generation and further catabolized into various SCFAs. This supports the notion that specific bacterial strains contribute to the breakdown of tryptophan, affecting the metabolic balance of this amino acid and its derivatives in the host.^[42]^ Additionally, findings from other studies on microbial metabolism of amino acids show that the conversion of tryptophan into pyruvate is a recognized process within certain bacterial species. The role of pyruvate as a central metabolic intermediate, involved in pathways like glycolysis and fermentation, ties into how gut bacteria may influence the balance of tryptophan metabolism, further lowering its availability relative to pyruvate.^[43]^ In our study, the species *Eubacterium callanderi* could decrease UC risk through decreasing tryptophan to pyruvate ratio. Studies have shown that tryptophan metabolism through the kynurenine pathway increases during inflammation, which is a hallmark of UC. Elevated levels of kynurenine metabolites, such as kynurenic acid, are associated with more severe endoscopic inflammation and poor clinical outcomes in UC patients.^[44]^ Our research suggested that gut microbiota plays a critical role in tryptophan metabolism. Changes in the composition of gut bacteria, particularly in individuals with UC, affect tryptophan catabolism, which may worsen gut inflammation by influencing pathways like the aryl hydrocarbon receptor signaling, known to regulate immune responses and maintain gut barrier function.^[45]^

The species *CAG-590 sp000431135*, belonging to the phylum *Firmicutes*, class *Clostridia*, order *Lachnospirales*, family *Lachnospiraceae*, and genus *CAG-590*, is an uncultured species defined from metagenome-assembled reference genomes.^[12]^ Unfortunately, there isn’t readily available detailed information on this particular species in popular biological databases or literature, despite our study revealing that species *CAG-590 sp000431135* was associated with an increased risk of UC, mediated through increased mannitol/sorbitol levels. However, the association between elevated levels of sorbitol and mannitol with an increased risk of UC aligns with existing theories on gut permeability and inflammation. Mannitol and sorbitol are types of polyols (sugar alcohols) that can be poorly absorbed in the small intestine, especially in individuals with gastrointestinal disorders such as irritable bowel syndrome or UC.^[46]^ This poor absorption can lead to increased intestinal permeability, often referred to as “leaky gut,” which is thought to contribute to the pathogenesis of UC. Increased intestinal permeability allows larger, potentially inflammatory molecules to pass through the gut lining and activate the immune system, leading to chronic inflammation, which is a hallmark of UC.^[47]^ In addition, the bacterial fermentation of mannitol and sorbitol can produce gases and other byproducts that further irritate the intestinal lining, exacerbating symptoms of UC.^[48]^

Our research offers several strengths. First, the study utilizes MR analysis, a robust statistical method that helps infer causal relationships, minimizing confounding factors and reverse causality. Second, the research draws from publicly available GWAS involving large sample sizes. It includes over 17,000 cases and 883,000 controls, enhancing the study’s statistical power and reliability. Third, the study explores the microbiota-gut link using forward and reverse MR analyses and also investigates plasma metabolites’ mediation role, allowing for a more nuanced understanding of the microbiota-gut health relationship. Last, the use of meta-analyses, sensitivity analyses (e.g., MR-Egger, weighted median, MR-PRESSO), and genetic correlation testing via LDSC strengthen the credibility of the results. These methods detect and account for pleiotropy and heterogeneity, ensuring that the findings are stable and valid.

Evaluating our findings requires an awareness of the study’s limitations. While MR techniques effectively reduce confounding and reverse causality, they remain vulnerable to potential biases. For instance, population stratification can introduce bias if there are systematic differences in allele frequencies between subpopulations. We mitigated this by focusing on individuals of European ancestry. However, the absence of comprehensive data limited our ability to perform stratified analyses by age and gender, constraining our ability to examine potential differences within demographic groups. Additionally, due to the extensive number of exposures investigated in our study, manually filtering out potentially pleiotropic SNPs through such resources is not feasible. To address this, we conducted MR analyses using outcome data from multiple independent databases. By performing meta-analyses to combine results from these different sources, we aimed to enhance the robustness and reliability of our findings.^[49]^ If any of our analyses indicated pleiotropy, we excluded those specific results from the meta-analysis.^[21]^ Finally, the product of coefficients method for mediation analysis assumes no unmeasured confounding, which may not always hold, particularly in complex biological systems. It also presumes linear relationships between the exposure, mediator, and outcome, potentially oversimplifying nonlinear biological interactions. Future research should focus on strengthening data collection to enhance representativeness and conducting detailed stratified analyses to explore differences and trends among various groups.

## 5. Conclusion

In concluding this study on the role of gut microbiota and plasma metabolites in UC through MR, our findings elucidate significant pathways through which the microbiota influences UC. Our results suggest that targeted microbial species such as *Clostridium S felsineum* and *Eubacterium callanderi* exert significant indirect effects on UC through mediation by specific metabolites like carnitine and tryptophan derivatives. These insights not only advance our understanding of the microbiological underpinnings of UC but also highlight potential therapeutic targets for modulating disease through dietary or probiotic interventions aimed at altering gut microbial composition. Future research on UC should delve deeper into the complex interactions between gut microbiota and plasma metabolites across diverse populations to expand the generalizability of results.

## Acknowledgments

The authors express their gratitude toward all the participants and investigators who contributed to the GWASs involved in the present study by generously sharing the summary-level data.

## Author contributions

**Conceptualization:** XuWen Zheng.

**Data curation:** JinNan Yin.

**Formal analysis:** JinNan Yin.

**Investigation:** JinNan Yin.

**Methodology:** XuWen Zheng.

**Project administration:** JinNuo Fan.

**Resources:** JinNuo Fan.

**Software:** XuWen Zheng.

**Supervision:** Ying Chu.

**Validation:** Ying Chu.

**Visualization:** XuWen Zheng.

**Writing – original draft:** XuWen Zheng.

**Writing – review & editing:** JinNan Yin, Ying Chu.

## Supplementary Material


